# Acceptance of the WHO Surgical Safety Checklist among surgical personnel in hospitals in Guatemala city

**DOI:** 10.1186/1472-6963-12-169

**Published:** 2012-06-21

**Authors:** Juan J Delgado Hurtado, Xavier Jiménez, Marco A Peñalonzo, Claudia Villatoro, Sandra de Izquierdo, Mónica Cifuentes

**Affiliations:** 1 Francisco Marroquín University. School of Medicine, Guatemala City, Guatemala; 2 Endocrine surgery, Francisco Marroquín University. School of Medicine, Guatemala City, Guatemala; 3 Anesthesiology department, Hospital General San Juan de Dios, Guatemala City, Guatemala

## Abstract

**Background:**

Studies have highlighted the effects the use of the WHO Surgical Safety Checklist can have on lowering mortality and surgical complications. Implementation of the checklist is not easy and several barriers have been identified. Few studies have addressed personnel’s acceptance and attitudes toward the WHO Surgical Safety Checklist. Determining personnel’s acceptance might reflect their intention to use the checklist while their awareness and knowledge of the checklist might assess the effectiveness of the training process.

**Methods:**

Through an anonymous self- responded questionnaire, general characteristics of the respondents (age, gender, profession and years spent studying or working at the hospital), knowledge of the WHO Surgical Safety Checklist (awareness of existence, knowledge of objectives, knowledge of correct use), acceptance of the checklist and its implementation (including personal belief of benefits of using the checklist), current use, teamwork and safety climate appreciation were determined.

**Results:**

Of the 147 surgical personnel who answered the questionnaire, 93.8% were aware of the existence of the WHO Surgical Safety Checklist and 88.8% of them reported knowing its objectives. More nurses than other personnel knew the checklist had to be used before the induction of anesthesia, skin incision, and before the patient leaves the operating room. Most personnel thought using the WHO Surgical Safety Checklist is beneficial and that its implementation was a good decision. Between 73.7% and 100% of nurses in public and private hospitals, respectively, reported the checklist had been used either always or almost always in the general elective surgeries they had participated in during the current year.

**Conclusions:**

Despite high acceptance of the checklist among personnel, gaps in knowledge about when the checklist should be used still exist. This can jeopardize effective implementation and correct use of the checklist in hospitals in Guatemala City. Efforts should aim to universal awareness and complete knowledge on why and how the checklist should be used.

## Background

In 2004, the global volume of major surgery was between 187.2 and 281.2 million cases per year [1]. Major morbidity complicates 3-16% of all inpatient surgical procedures in developed countries, with death rates of about 0.4-0.8%. In developing countries, death rates are estimated to be between 5-10% for major surgeries [1]. Mortality related to general anesthesia is as high as 0.6% in parts of Sub-Saharan Africa [2]. According to this report, half of the adverse events were thought to be preventable.

Due to the high volume of surgical procedures and high complication rates, the World Health Organization (WHO) launched the Save Surgery Saves Lives Initiative in 2007 [3]. The goal of this initiative was to “improve the safety of surgical care around the world by defining a core set of safety standards that can be applied in all WHO Member States” [3]. Four areas were identified for improvement: surgical site infection prevention, safe anesthesia, safe surgical teams and measurement of surgical services. Four ways to improve safety were identified: defining a minimum set of uniform measures, providing information on the role and pattern of surgical safety, identifying a set of surgical safety standards (compiled in a checklist), and evaluating and disseminating the proposed checklist [3]. The proposed surgical safety checklist was tested in a prospective pilot study in eight hospitals (in eight cities) that represented a variety of economic circumstances. The death rate decreased from 1.5% before the checklist was implemented to 0.8% afterward. Inpatient complications also decreased from 11% to 7% [4].

In 2008, the Surgical Safety Checklist was released along with implementation material. Since then, more than 3,900 hospitals in more than 122 countries worldwide have registered as participating hospitals who intend to introduce the WHO Surgical Safety Checklist in their operating rooms. Of these, more than 1,800 hospitals have reported using it routinely in at least one operating theater [5,6]. In Guatemala in 2010, 12 hospitals were committed to implementing it in their operating theaters [7].

To have the kind of impact reported in the pilot study [4], implementation of the checklists has to be effective. Active leadership, deliberate enrollment, extensive discussion and training, piloting, multidisciplinary communication, real-time coaching, ongoing feedback and regular audits are characteristics that determine highly effective implementation processes [8-10]. Operating room personnel must understand why the checklist should be used and know how it should be used [9]. Some barriers that can prevent correct implementation are unfamiliarity and embarrassment, hierarchy, timing of the checks, duplications with existing processes, lack of communication and modification of the checklist [10]. Specialists have considered that implementing checklists in health care is not as easy as had been thought. Reasons for this are social, cultural, and operational. For example, some clinicians might view standardization as a limitation to their clinical judgment [11,12].

Few studies have addressed differences in opinion of surgical personnel toward the WHO Surgical Safety Checklist [10]. Some have suggested junior doctors may have an important role as agents of change [13]. Others have described substantial support for the checklist from anesthesiologists and nurses [10].

A strong factor that affects clinicians’ involvement in patient safety behavior is their belief about whether engaging in a behavior improves patient safety and whether the individual has sensed improved patient safety resulting from the behavior [14]. Describing personnel’s acceptance and attitudes toward the Surgical Safety Checklist is important since it might reflect their intent on using this instrument, facilitating or impeding its implementation. Determining personnel’s awareness and knowledge of the checklist might help us assess the effectiveness of the training process. This study attempted to determine operating room personnel’s knowledge and acceptance of the checklist (including personal believes), one year after it was introduced in the surgical setting.

## Methods

We conducted a descriptive study during August and September 2011 in three hospitals located in Guatemala City, one year after the Surgical Safety Checklist (SSC) was implemented. Two of the selected hospitals are public teaching hospitals and the other is privately owned. The study was approved by the Research Department of the Francisco Marroquín University as well as by each participating hospital’s research and their ethics committees.

The questionnaire explored general characteristics of the respondents (age, gender, profession and years spent studying or working at the hospital), knowledge of the WHO SSC (awareness of existence, knowledge of objectives, knowledge of correct use), acceptance of its implementation (including personal belief of benefits of using the checklist), current use, teamwork and safety climate appreciation. To determine current teamwork and safety climate appreciation, the Safety Attitude Questionnaire (SAQ) OR version [15], developed by the Center for Health Care Safety and Quality of the University of Texas was translated into Spanish and slightly modified. Permission to use, translate and modify this questionnaire was granted by the Center for Health Care Safety and Quality. The mean SAQ score was calculated on a scale of 1 to 5, with 5 representing the highest safety attitude and 1 representing the lowest.

The survey was pre-tested through two focus groups with surgical gynecology nurses at one of the hospitals in which checklist implementation was underway, but in a hospital not included in this study. Focus groups the participants were asked how comprehensive the questions were and to suggest any modification to the questions to improve their comprehension. On the basis of the pretesting, modifications were made to a few questions and response categories.

The anonymous self-administered questionnaire was distributed to surgical personnel (nurses, anesthesiologists, anesthesiology residents, and surgery residents) during staff meetings or academic activities. Before responding to the survey, they had to read and sign an informed consent form. The first part of the questionnaire explored the awareness of the existence of the WHO SSC. Persons who responded being aware of the existence of the SSC were further questioned on their knowledge of objectives, knowledge of correct use, acceptance of its implementation, and current use of the SSC. The ‘current teamwork and safety climate appreciation’ part of the questionnaire was responded by all personnel.

We compared responses for the different personnel by using Z score. Epi Info version 3.5.3 was used for all calculations. Data are presented as relative and absolute values with standard deviation.

## Results

The survey was administered to 149 surgical personnel and of them, 147 responded it (98.66% response rate): 40 surgery residents, six anesthesiologists, 45 anesthesiology residents and 56 nurses (Table 1). Not all the personnel responded all of the questions.

**Table 1:** General characteristics of the sample

	**Mean age (years) (n = 141)**	**Gender (n = 144)**	**Years spent studying or working at the hospital**
**Surgery residents (40)**	28.1 ± 2.45	Male 61.5% (24)	2.5 ± 1.22
		Female 38.5% (15)	
**Anesthesiology residents (45)**	28.1 ± 2.16	Male 22.2% (10)	2.12 ± 1.17
		Female 77.8% (35)	
**Anesthesiologists (6)**	48.6 ± 16.59	Male 16.7% (1)	7.66 ± 4.5
		Female 83.3% (5)	
**Nurses (56)**	33.0 ± 9.34	Male 9.3% (5)	8.46 ± 7.11
		Female 90.7% (49)	

Of all respondents, 93.8% were aware of the existence of the WHO SSC (89.7% of surgery residents, 97.8% of anesthesiology residents, 100% of anesthesiologists and 92.9% of nurses) and of those, 88.8% reported knowing the objectives of the checklist (80% of surgery residents, 95.5% of anesthesiology residents, 100% of anesthesiologists, and 87.8% of nurses). The most common way respondents learned about the checklist was either through publicity at the hospital (46.9%) or training courses (28.6%); 6.8% learned of it at a conference. Overall, 95.6% of personnel had seen the checklist in the operating room (OR).

More nurses (84.6%) and anesthesiologists (83.3%) than surgery residents (64.7%) and anesthesiology residents (69.8%) knew the checklist had to be used at the three time points: before the induction of the anesthesia, before skin incision, and before the patient leaves the OR. Significantly more nurses than surgery residents knew that the checklist had to be used at the three time points (Z = 2.137, p = 0.032).

According to 73.7% of nurses working at the two public hospitals, the checklist had been used either always or almost always in the general elective surgeries they had participated in during the current year, whereas 100% of the nurses working at the private hospital reported that it had been used always or almost always. According to our respondents, the circulating nurse had been the person in charge of reading the items and assuring the use of the checklist in most of the surgeries in which it had been used (96.9%).

When questioned about their beliefs of the benefit of using the WHO SSC the different groups generally agreed that it is beneficial (Table 2). When personnel were asked if they had ever made an error in the surgical room that could have been prevented, 42.5% (17) of surgery residents, 63.2% (28) of the anesthesiology residents, 100% (6) of the anesthesiologists and 35.7% (20) of the nurses responded they had.

**Table 2:** Acceptance and beliefs of the benefit of using the Surgical Safety Checklist among surgical personnel

	**Surgery residents**	**Anesthesiology residents**	**Anesthesiologists**	**Nurses**
SSC^1^ stimulates communication? (yes)	80% (20)	88.6% (39)	100% (6)	92.3% (48)
(no)	11.4% (4)	11.4%(5)	0%(0)	5.8% (3)
SSC helps prevent errors? (yes)	88.6% (31)	93.2% (41)	100% (5)	84.3% (43)
(no)	8.6%(3)	6.8% (3)	0(0%)	15.7% (8)
Implementing the SSC was a good decision? (yes)	97.1% (34)	100% (44)	100% (6)	98% (49)
(no)	0%(0)	0%(0)	0%(0)	2%(1)
If you were undergoing surgery would you like the personnel use the SSC? (yes)	100%(35)	97.7% (43)	100% (6)	98% (50)
(no)	0%(0)	0%(0)	0%(0)	2%(1)
Using the SSC is? Easy or really easy (n = 107)	91.4% (32)	86.3% (38)	66% (4)	63.5% (33)

^1^SSC Surgical Safety Checklist.

The mean score for team work climate appreciation by respondents was 3.46, and the mean score for safety climate appreciation was 3.52.

## Discussion

This study shows high acceptance of the WHO SSC among surgery and anesthesiology residents, nurses and anesthesiologists. This is similar to a study in which 80.2% and 84.8% of clinicians thought the checklist improved operating room safety and communication after its implementation, respectively [16]. 78.6% thought the checklist helps prevent errors in the operating room [16]. Another study found that 97% of personnel thought adopting a time out checklist could prevent mistakes [17].

Although overall awareness of the checklist is high, reported knowledge of the objectives of the checklist is not as high. Awareness of the existence of the checklist is lower than what a study done in the UK found, in which 99% of senior theater staff of 238 hospitals responded having heard of the WHO SSC [18]. Our results suggest there is a gap in knowledge as to when the surgical safety checklist should be used. Fewer anesthesiologists, surgery residents, and anesthesiology residents than nurses knew the checklist had to be used before the induction of the anesthesia, before the skin incision, and before the patient leaves the OR. This could be a barrier to effective implementation and correct use of the checklist. Effective implementation, consistent and correct use of the checklist has been associated with team effort which requires complete knowledge of how the checklist has to be used and its goals [9,10]. A moderately effective implementation process was characterized by explaining why the checklist should be used, but failing to show how it should be implemented [9]. These results highlight the need for training focused on when and how the checklist has to be used. Training reinforcing why the checklist should be used is important since it is a predecessor for showing how it should be used [9]. Multidisciplinary communication, training, coaching and feedback could help improve inter personnel knowledge regarding this issue.

According to the personnel reports, the checklist is not being used consistently. Results suggest a difference in consistency of use of the checklist between the public hospitals and the private one. Implementation training and efforts might differ between hospitals explaining differences in reported compliance. However, further observational studies should be done to determine actual compliance with the checklist.

The team work and safety climate perception scores can be used as a measure of patient safety improvement after the implementation of tools such as the WHO SSC [16,19]. A study described an increase in teamwork and safety climate perception after the implementation of the WHO SSC [16]. A factor described for successful implementation is the encouragement and support of local measures of effectiveness [10]. To our knowledge, this score had not been determined in our setting before and can be used in training and further studies.

This study has important limitations, among them are: the differences on the number of participants in the subgroups; and the self-reported nature of the questionnaire. Further studies should determine the acceptance and use of the Surgical Safety Checklist among personnel, including surgeons.

## Conclusions

Although there is a high acceptance and adequate self-reported awareness of the WHO Surgical Safety Checklist among surgical personnel in Guatemala, there is a gap in knowledge as to when the surgical checklist has to be used. Efforts should aim to universal awareness and complete knowledge on why and how the checklist should be used.

Acceptance and knowledge are some of the factors that might determine compliance in using the checklist. There are a number of other variables that need to be further explored. A study that explores current compliance with the checklist directly through observation or indirectly through register review should be done. It would also be important to keep track, through different indicators, of the effects compliance with the SSC has on patient safety locally.

## Competing interests

The authors declare that they have no competing interests.

## Authors’ contributions

JJD participated in the design, acquisition of data, analysis and drafting of the manuscript. XJ and CV cooperated in the acquisition of data and critically reviewed the manuscript. MAP, SI and MC participated in the design of the study. All authors read and approved the final manuscript.

## Pre-publication history

The pre-publication history for this paper can be accessed here:

http://www.biomedcentral.com/1472-6963/12/169/prepub
